# An Unusual Presentation of Intramuscular Cysticercosis in a Vegetarian Patient: A Case Report

**DOI:** 10.7759/cureus.67166

**Published:** 2024-08-19

**Authors:** Dattatray Bhakare, Urva Dholu, Rahul Salunkhe, Swati Bhakare, Pratik T Gundecha

**Affiliations:** 1 Department of Orthopaedics, Dr. D. Y. Patil Medical College, Hospital and Research Centre, Dr. D. Y. Patil Vidyapeeth (Deemed to be University), Pune, IND; 2 Department of Obstetrics and Gynaecology, Dr. D. Y. Patil Medical College, Hospital and Research Centre, Dr. D. Y. Patil Vidyapeeth (Deemed to be University), Pune, IND

**Keywords:** endemic, vascular thrombosis, flexor digitorum profundus, vegetarian, intramuscular

## Abstract

Cysticercosis is a parasitic infection. It can involve any tissue in the body, the brain being the most common site. Intramuscular cysticercosis is rare, and few cases have been reported. Here, we are reporting a case of incidental finding of intramuscular cysticercosis in a 22-year-old male having a purely vegetarian diet presented with pain, swelling, and tingling sensation in the forearm without any other systemic involvement after taking Albendazole for unrelated reasons. Diagnosis of intramuscular cysticercosis remains a challenge and should be considered in the differential diagnosis of intramuscular swelling, especially in endemic areas.

## Introduction

Cysticercosis is a parasitic infestation of the body caused by cestodes, the pork tapeworm, *Taenia solium* [1]. Cysticercosis is a global health issue, yet it is more prevalent in developing nations because of limited access to sanitation facilities as well as frequent contact between animals and humans, particularly pigs. Among immunocompetent individuals, it ranks as the most widespread parasitic infection affecting the nervous system [2]. Both vegetarians and non-vegetarians may be infected. Depending on the pathogenesis, three different types of clinical manifestations for the muscular form have been described: the myalgic type; the mass-like, pseudotumor, or abscess-like type; and the rare pseudo hypertrophic type [3]. The occurrence of cysticercosis exclusively within muscles is rare. When it does occur, it is often overlooked or underreported due to the high level of suspicion required for diagnosis and its status as a neglected disease [2]. Unlike neurocysticercosis, isolated muscle involvement usually does not result in death [4].

## Case presentation

**Figure 1:**
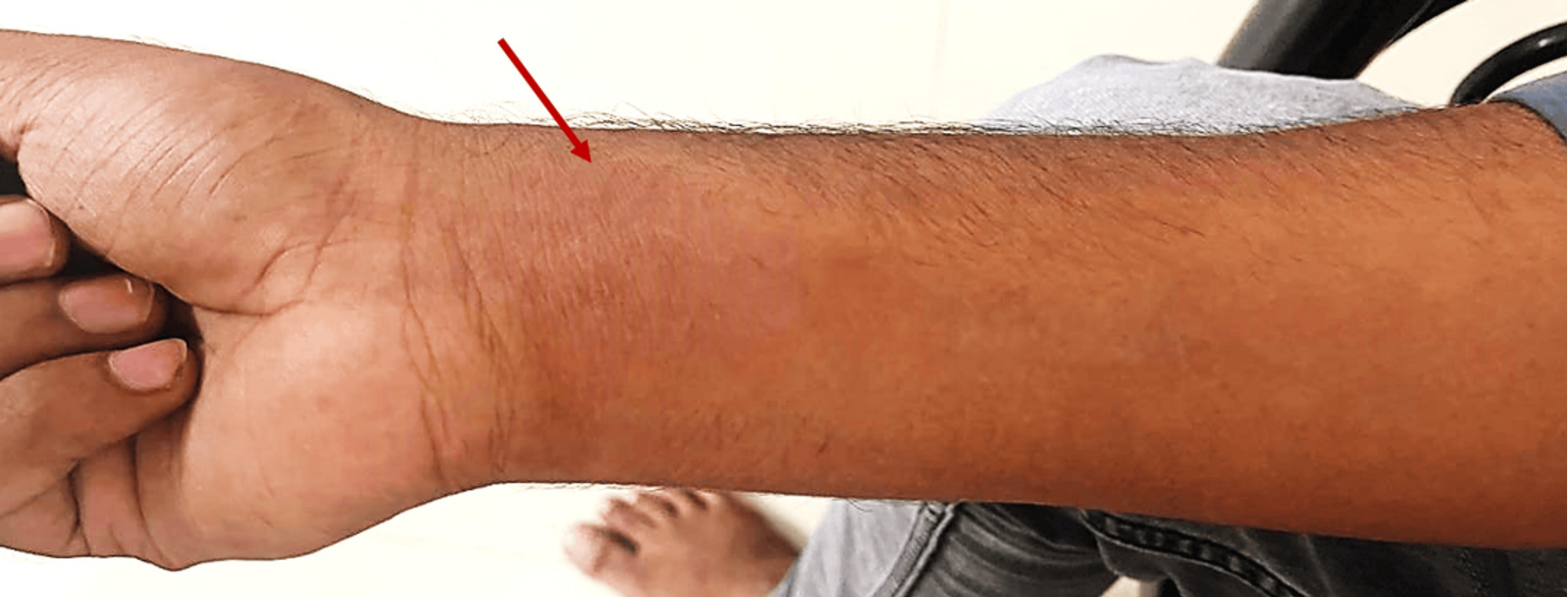
Clinical picture showing swelling and redness over the flexor aspect of the right forearm.

A 22-year-old male patient developed pain, swelling, and tingling sensation in the flexor aspect of the right forearm and came to our Orthopaedic OPD for the same complaints. On examination, there was swelling, redness, stretched pain, and deep tenderness over the flexor aspect of the right forearm (Figure 1).

**Figure 2:**
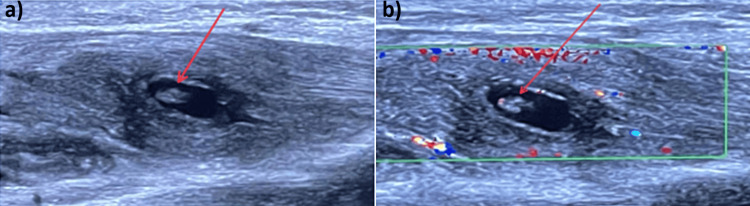
(a) USG forearm. (b) USG arteriovenous (A-V) Doppler, which was suggestive of a well-defined, thick-walled cystic lesion measuring 10 × 5 cm noted in the intramuscular plane (flexor digitorum profundus) in the anterior aspect of the right forearm with a hyperechoic focus within scolex.

The patient inadvertently took a single dose of 600 mg of Albendazole for reasons unrelated to treating a parasitic infection. He has no history of traumatic injury to the right forearm, and he is a pure vegetarian. The patient had no other systemic complaints. Initially, there was the consideration of a preliminary diagnosis of vascular thrombosis, compartment syndrome, and tumor presentation. To rule out vascular thrombosis and compartment syndrome, USG with arteriovenous (A-V) Doppler was advised [5]. The imaging findings suggestive of a cysticercosis infection were reported, showing a well-defined, thick-walled cystic lesion measuring 10 × 5 cm in the intramuscular plane (flexor digitorum profundus) in the anterior aspect of the right forearm. The lesion contained a hyperechoic focus within, which may represent a scolex (Figure 2).

**Figure 3:**
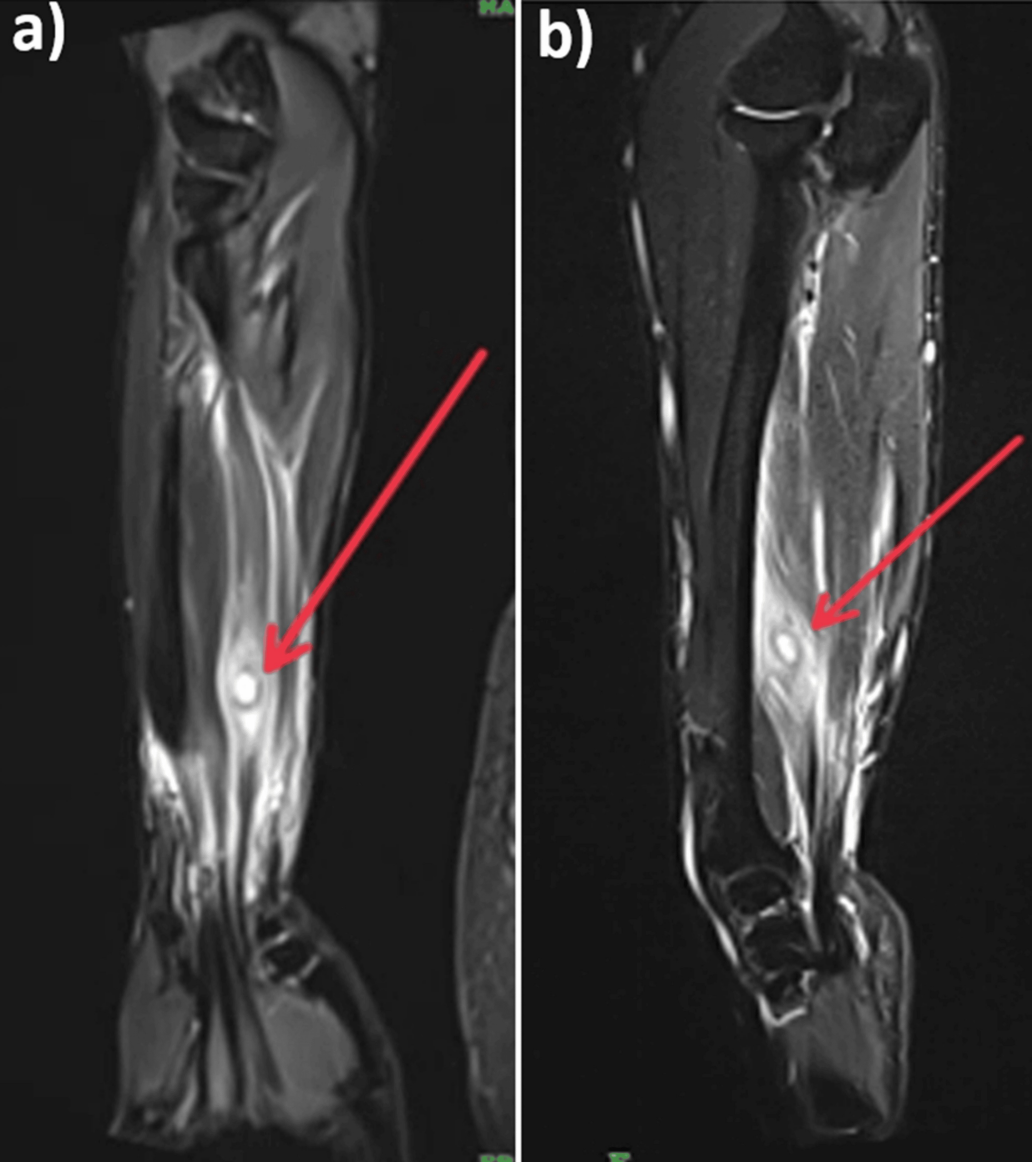
MRI of the right forearm. (a) Coronal cut. (b) Sagittal cut. MRI findings came out to be suggestive of a well-defined oval cystic lesion in the flexor digitorum profundus muscle showing scolex, partial calcification of the wall and a focal defect in the wall.

The gold standard method of investigation of cysticercosis is MRI. The patient was advised to have an MRI of the right forearm. MRI findings came out to be suggestive of a well-defined oval cystic lesion in the flexor digitorum profundus muscle showing scolex, partial calcification of the wall, and a focal defect in the wall. Extensive surrounding myofascial and subcutaneous edema were noted in the forearm. It is most likely due to an intramuscular cysticercosis (Figure 3).

The patient’s diagnosis was confirmed and was treated conservatively with non-steroidal anti-inflammatory drugs.

## Discussion

The parasitic disease known as cysticercosis is brought on by the *Taenia solium* tapeworm larval stage. In regions with inadequate sanitation and hygiene standards, it is a serious public health hazard. Intramuscular cysticercosis is a form of cysticercosis where the larval stage of the *Taenia solium* tapeworm infects and forms cysts within the muscles. While cysticercosis can affect various tissues and organs, intramuscular cysticercosis specifically involves the skeletal muscles and is relatively common in regions where the parasite is endemic. In intramuscular cysticercosis, the larvae form cysts within the muscle tissue. These cysts can cause a range of symptoms depending on their location and size. Common symptoms may include muscle pain, weakness, swelling, tingling sensation, and palpable lumps or nodules in the affected muscle. In some cases, individuals may not experience any symptoms, especially if the cysts are small or located in less noticeable areas.

Ultrasonography, or USG, is a helpful diagnostic technique for suspected cysticercosis. It is inexpensive, easily accessible, and non-invasive [6]. One diagnostic feature of intramuscular and subcutaneous cysticercosis is the characteristic appearance of a cyst with an echogenic scolex positioned centrally [7,8,9]. Tests, such as immunodiagnostics, are a crucial diagnostic tool for *Taenia solium* cysticercosis, the disease it causes. Numerous methods, including indirect hemagglutination assays, indirect fluorescent antibody assays, enzyme-linked immunosorbent assay (ELISA), complement fixation tests, radioimmunoassay, dot blot, immunoelectrophoresis, and enzyme-linked immunoelectrotransfer blot (EITB), have been used to diagnose human cysticercosis immunologically [10]. For people with living cysticerci, therapy is recommended. Praziquantel (50 mg/kg for 15 to 30 days) or Albendazole (10 to 15 mg/kg for 10 to 15 days) are the most effective treatments for it. Only five cases of this single unilocular or multilocular cyst have been documented in the literature. They occurred in the flexor digitorum profundus, psoas, extensor carpi ulnaris, biceps brachii muscle, and tendoachilles tendon sheath. This scarcity of reported cases underscores the rarity of isolated intramuscular cysticercosis and suggests that it may be underdiagnosed or underreported due to its uncommon presentation. Research has shown that while the larvae develop inside the cyst, they might not become very antigenic until the host reaction or chemotherapy gradually kills the cyst, causing noticeable inflammation and pericystic edema. This case report describes an atypical presentation of intramuscular cysticercosis in a young, asymptomatic male with no history of traumatic injury to the affected forearm. This presentation contrasts with typical cases that may involve neurological symptoms or manifestations in other organs. The patient's vegetarian diet adds another layer of uniqueness to the case, as cysticercosis is often associated with the consumption of contaminated pork. This raises questions about alternative routes of infection and underscores the versatility of the parasite in adapting to different host environments. Additionally, the localization of the cyst within the flexor digitorum profundus muscle of the forearm is unusual, as cysticercosis typically affects tissues such as the brain, eyes, or subcutaneous tissues. The rarity of intramuscular cysticercosis poses diagnostic challenges, as clinicians may not immediately consider it in the differential diagnosis of muscle pain and swelling. Furthermore, the need for specialized imaging modalities, such as MRI, to confirm the diagnosis adds to the complexity of identifying and managing these cases. Despite its rarity, intramuscular cysticercosis remains clinically significant due to its potential to cause symptoms such as pain, swelling, and functional impairment in affected individuals. Additionally, delayed diagnosis and treatment can lead to complications, such as inflammation and pericystic edema, as observed in the discussed case. Highlighting the rarity of cases like the one presented underscores the importance of raising awareness among healthcare professionals about the diverse clinical presentations of cysticercosis. Increased awareness can lead to earlier recognition and suitable management of such cases, ultimately improving patient outcomes as well as lowering the burden of this neglected tropical disease. By emphasizing the rarity of the case within the broader context of cysticercosis, we would like to emphasize the need for vigilance and comprehensive diagnostic evaluation when encountering atypical presentations of parasitic infections, particularly in regions where cysticercosis is endemic.

## Conclusions

This case report sheds light on the rarity of intramuscular cysticercosis, emphasizing its potential for underdiagnosis or misdiagnosis due to its uncommon presentation. The case underscores the significance of considering parasitic infections, such as cysticercosis, in the differential diagnosis of muscle swellings, particularly in regions where the disease is endemic. Furthermore, the unique aspects of this case highlight the need for heightened vigilance and comprehensive diagnostic evaluation when encountering unusual presentations of parasitic diseases. This case also emphasizes the value of early diagnosis and adequate management of cysticercosis and raises awareness among healthcare professionals.
